# Fabric Phase Sorptive Extraction Prior to HPLC–DAD Determination of Monomers Released from Dental Resin Composites in Dental Wastewater

**DOI:** 10.3390/molecules31213873

**Published:** 2026-10-27

**Authors:** Ifigeneia Tragianni, Pavlos Voutsas, Natasa Kalogiouri, Abuzar Kabir, Petros Mourouzis, Victoria Samanidou

**Affiliations:** 1 Laboratory of Analytical Chemistry, School of Chemistry, Aristotle University of Thessaloniki, 54124 Thessaloniki, Greece; itragia@chem.auth.gr (I.T.); pvoutsaa@chem.auth.gr (P.V.); kalogiourin@chem.auth.gr (N.K.); 2 Department of Chemistry and Biochemistry, International Forensic Research Institute, Florida International University, Miami, FL 33199, USA; akabir@fiu.edu; 3 Division of Operative Dentistry, Faculty of Dentistry, School of Dental Tissues Pathology and Therapeutics, Aristotle University of Thessaloniki, 54124 Thessaloniki, Greece; pmourouzis@dent.auth.gr

**Keywords:** dental resin composites, fabric phase sorptive extraction (FPSE), high-performance liquid chromatography (HPLC), dental wastewater, BPA, TEGDMA, UDMA, Bis-GMA

## Abstract

A novel fabric phase sorptive extraction method (FPSE) was developed for the detection of monomers released during dental restorative procedures, which are subsequently discharged into the environment through dental unit drainage systems. The presence of Bisphenol-A (BPA), Triethylene glycol dimethacrylate (TEGDMA), Urethane dimethacrylate (UDMA) and Bisphenol A-glycidyl methacrylate (Bis-GMA), released from dental resin composites in dental wastewater, was monitored using high-performance liquid chromatography coupled to diode array analysis (HPLC–DAD). FPSE was optimized with respect to the following significant parameters: (a) membrane type, (b) membrane size, (c) extraction time, (d) stirring during extraction, (e) sample volume, (f) ionic strength, (g) elution solvent, (h) elution time, and (i) agitation during elution. For HPLC–DAD analysis, the mobile phase constituted of acetonitrile–water, and a gradient elution program was followed. The detection was carried out at 220 nm. The method was validated in terms of linearity, sensitivity, selectivity, accuracy, and precision, showing satisfactory results. The limits of detection (LOD) and quantification (LOQ) were equal to 0.015 ng/μL and 0.05 ng/μL, respectively, for BPA, TEGDMA, and Bis-GMA, and 0.04 ng/μL and 0.13 ng/μL, respectively, for UDMA. The method demonstrated satisfactory precision, with relative standard deviation (RSD) values lower than 7.7% for intra-day and lower than 9.5% for inter-day studies. Relative recoveries ranged from 93.9% to 100.8% (intra-day) and from 92.0% to 101.7% (inter-day) for all target analytes. The developed FPSE–HPLC–DAD method was applied to the filtered/dissolved fraction of a real dental wastewater sample after centrifugation and filtration.

## 1. Introduction

Dental resin composites (DRCs) are widely used in modern restorative dentistry, replacing dental amalgam. Dental amalgam represented a milestone in restorative dentistry for many decades; however, due to its high mercury content and its inability to meet modern aesthetic requirements, it has been replaced by new materials. The Minamata Convention on Mercury also played a significant role in limiting the clinical use of amalgam [1]. DRCs offer numerous advantages, including excellent aesthetics and superior mechanical properties [2]. Nevertheless, there are concerns regarding the release of their components and their potential impact on both human health and the environment [3,4].

Despite the passage of decades, the basic structure of DRCs remains as follows: the organic resin matrix, the inorganic fillers, and the coupling agent, which is essential for binding them together. The organic resin matrix consists of monomers that form a stable network. Bis-GMA and UDMA are the primary monomers, used individually or in combination, to synthesize the matrix. TEGDMA is used as a diluent monomer, regulating the fluidity of the final material [5,6]. However, there are concerns regarding the potential release of these monomers from DRCs, as well as of BPA, which is not a direct component of the materials but an impurity of the synthesis of Bis-GMA [7].

The release of monomers from DRCs can be studied at two levels: at a patient level and at an environmental level. The release of components into the oral cavity results from incomplete polymerization and long-term degradation of the material, which is influenced by saliva, chewing, and thermal and chemical changes related to diet [8]. During clinical dental procedures, such as the fitting or removal of a composite resin restoration, the organic matrix is degraded through the grinding of the material, while at the same time resin microparticles are produced, promoting the release of organic monomers. These materials together with patients’ saliva are removed from the oral cavity using a suction device, discharged into wastewater, and, finally, released into the environment [9,10].

In recent decades, many studies have focused on the release of these monomers from DRCs into various solvents as a part of an in vitro simulation of the oral cavity. In the majority of these studies, dental materials were immersed in organic solvents, such as methanol [11] and 75% ethanol [12,13], or aqueous media, such as deionized water [14] and artificial saliva [15], for varying periods of time. Then, the immersion media were directly analyzed in order to determine the concentrations of the released monomers. The most applied instrumental techniques are high-performance liquid chromatography (HPLC), liquid chromatography–mass spectrometry (LC–MS), and gas chromatography (GC) [16,17,18].

However, studying the release of these monomers in more complex sample matrices, such as biological fluids or dental wastewater, also requires appropriate sample preparation. Sample preparation is one of the most time-consuming and challenging steps in an analytical procedure, yet it is crucial for the subsequent instrumental analysis. Due to green analytical chemistry (GAC) and the higher demands for the efficiency and practicality of analytical methods, newly developed extraction techniques are replacing the classical liquid–liquid extraction (LLE) and solid-phase extraction (SPE) techniques, shifting toward microextraction [19]. Microextraction techniques offer several advantages, including minimal solvent and sample consumption, reduced sample preparation time, high extraction efficiency, and compliance with the principles of GAC. Various microextraction techniques have been applied for the extraction and determination of BPA and resin monomers, including capsule phase microextraction (CPME) [20], solid-phase microextraction (SPME) [21,22], fabric phase sorptive extraction (FPSE) [23,24], liquid–liquid microextraction (LLME) [25], dispersive liquid–liquid microextraction (DLLME) [26], and stir bar sorptive extraction (SBSE) [27].

FPSE was proposed in 2014 by Kabir and Furton as a simple, versatile, and green sample preparation technique [28]. In this microextraction technique, the extraction of target analytes is carried out using a thin, permeable, and flexible fabric substrate coated with a porous, sponge-like, sol–gel hybrid inorganic–organic sorbent, enabling rapid mass transfer. The covalent bonding between the fabric substrate and the sol–gel sorbent coating provides the FPSE medium with high chemical and mechanical stability, allowing its application over a wide pH range and the use of organic solvents, while maintaining excellent reusability. Regarding the extraction stage, the technique can be directly applied to complex sample matrices without prior sample pretreatment, thereby shortening the overall analytical workflow and minimizing the risk of analyte loss. The elution of the target analytes requires only minimal amounts of organic solvents, in accordance with the principles of GAC [29].

Regarding the study of the release of resin monomers from DRCs into the environment, and specifically in dental wastewater, analytical techniques are limited [10,30].

Although the release of resin-based composite components has been widely investigated in artificial saliva, aqueous media, organic solvents, and biological matrices, analytical studies focusing specifically on dental wastewater remain limited. This matrix is relevant because composite adjustment, finishing, polishing, or removal procedures may generate resin-containing particles and dissolved organic compounds that are evacuated through dental unit suction systems. Therefore, there is a need for a simple, sensitive, and environmentally friendly sample-preparation method suitable for the determination of resin-derived monomers in dental wastewater. The novelty of the present work lies in the development and validation of an FPSE–HPLC–DAD method for the simultaneous determination of BPA, TEGDMA, UDMA, and Bis-GMA in this matrix, using low sample and solvent volumes and a reusable extraction medium.

## 2. Results and Discussion

### 2.1. Optimization of the FPSE Procedure

To develop an effective FPSE protocol, it is necessary to optimize the parameters that affect extraction efficiency. In this study, the initial FPSE protocol used as a starting point for the optimization was as follows: first, the FPSE membrane was activated in 2 mL of methanol and acetonitrile (MeOH, ACN) (50:50 *v*/*v*) for 5 min and rinsed with 2 mL HPLC-grade water (H_2_O) for 5 min. It was then immersed in 1 mL of water sample spiked with the target analytes, at a concentration of 1 ng/μL, and extraction was performed under moderate magnetic stirring for 20 min. After extraction, the membrane was rinsed with HPLC-grade H_2_O, air-dried for 3 min, and the elution was conducted in 1 mL MeOH for 10 min without stirring. The initial dimensions of the FPSE membrane were 1.0 cm^2^ (1.0 cm × 1.0 cm). For the optimization, the one-factor-at-a-time (OFAT) technique was applied, with the optimal condition for each parameter selected according to the % absolute recoveries of each analyte, as described in Section 3.6.

The selection of the optimal FPSE membrane is a critical factor affecting the extraction performance and is the first parameter that should be considered when developing an FPSE method. Five different sorbent coatings on cellulose substrate were evaluated: sol–gel Si-PTHF, sol–gel Ti-PTHF, sol–gel Ti-PEG300, sol–gel Ti-CW 20M, and sol–gel Ti-PCAP-PDMS-PCAP. The sol–gel Si-PTHF membrane has a silica-based sol–gel network, while all the other membranes have a titanium-based sol–gel network, depending on the sol–gel precursor used during the preparation of the FPSE membrane. The titanium-based sol–gel membranes display advantages over conventional silica-based networks, such as higher chemical and mechanical stability. Accordingly, four titanium-based membranes were evaluated, whereas only one silica-based membrane was tested. The aim was not to perform a systematic comparison between silica- and titanium-based coatings, but to identify the most suitable membrane among the available FPSE media. Based on the results presented in Table 1, the sol–gel Si-PTHF membrane was the most suitable for the extraction of BPA, TEGDMA, UDMA, and Bis-GMA, as it exhibited the highest recovery values for all four compounds. The sol–gel Ti-PCAP-PDMS-PCAP membrane did not show extraction capability for the target analytes. This may be attributed to its non-polar coating, in contrast to the other membranes evaluated.

**Figure 1:**
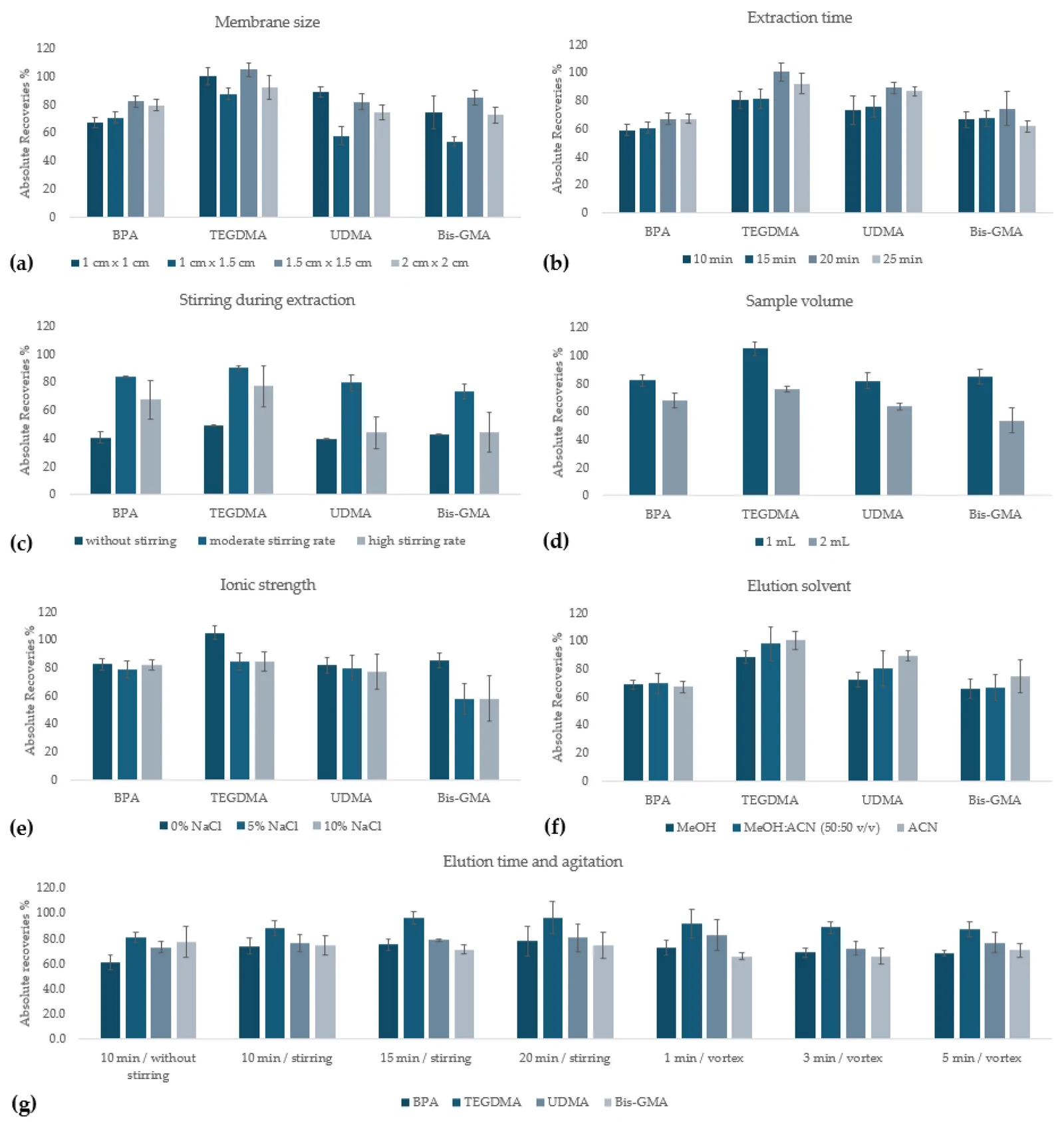
Optimization of (**a**) membrane size, (**b**) extraction time, (**c**) stirring during extraction, (**d**) sample volume, (**e**) ionic strength, (**f**) elution solvent, and (**g**) elution time and agitation.

An advantage of the FPSE technique is the ability to select the appropriate size of the FPSE membrane. As a part of the optimization process, four different sizes of FPSE membrane were evaluated: 1 cm × 1 cm (1 cm^2^), 1 cm × 1.5 cm (1.5 cm^2^), 1.5 cm × 1.5 cm (2.25 cm^2^), and 2 cm × 2 cm (4 cm^2^). According to the results presented in Figure 1a, the 1.5 cm × 1.5 cm (2.25 cm^2^) dimension was selected as optimal, showing satisfactory recoveries for all target analytes while also allowing easy handling during the extraction procedure.

For the optimization of the extraction time, four extraction times were examined: 10, 15, 20, and 25 min. As shown in Figure 1b, the recoveries of the target monomers increased at 20 min, compared with the shorter extraction times. At 25 min, a decrease in recoveries was observed, particularly for TEGDMA, UDMA, and Bis-GMA. This decrease may be attributed to partial desorption of the analytes from the FPSE membrane back into the sample during prolonged extraction. However, this mechanism could not be confirmed based on the available data. Therefore, 20 min was chosen as the optimal extraction time. During the extraction step, stirring of the sample enhances the extraction kinetics and facilitates mass transfer from the sample to the FPSE membrane. The effect of stirring was also investigated by examining the following three cases: absence of stirring, moderate stirring rate, and high stirring rate. The stirring speed was set to positions 2 (moderate stirring) and 6 (high stirring) on the stirrer’s 1–10 scale. Based on the experimental results shown in Figure 1c, moderate stirring rate was chosen as optimum for the extraction step. The absence of agitation hinders the mass transfer, while higher stirring rate leads to lower recoveries, while simultaneously causing partial damage to the membrane, reducing its reusability.

The effect of the sample volume on the extraction performance was evaluated at 1 and 2 mL. Smaller sample volumes were not evaluated, as 1 mL was considered a sufficiently small and practical volume for the analysis of aqueous environmental samples. According to the results (Figure 1d), increasing the sample volume hindered the extraction of the target compounds, possibly due to the reduced interaction between the target compounds and the FPSE membrane, as the compounds are dispersed over a larger volume. Therefore, 1 mL was selected as the working sample volume. However, intermediate volumes were not evaluated. This experiment was performed to evaluate the effect of sample volume on extraction efficiency and was not intended to establish a preconcentration factor.

The effect of ionic strength was also investigated as an optimization parameter by adding 5% and 10% (*w*/*v*) NaCl to the samples. On the one hand, increasing the ionic strength can induce a salting-out effect, leading to higher extraction efficiency. On the other hand, it may also increase the solution viscosity, slowing down the mass transfer and reducing extraction efficiency. As shown in Figure 1e, the addition of NaCl hindered the extraction, particularly for TEGDMA and Bis-GMA. Therefore, in the optimized protocol, NaCl was not added to the sample.

Sol–gel coating technology confers high chemical stability on the FPSE membrane, allowing exposure to any organic solvent or mixtures of organic solvents. Hence, for the final step of elution, the following elution solvents were assessed: MeOH, ACN, as well as an MeOH: ACN mixture (50:50 *v*/*v*). ACN was chosen as the optimal elution solvent, as it provided the highest recovery values, while avoiding the preparation of a solvent mixture (Figure 1f).

The effect of agitation and time on the elution step was also investigated. Two agitation types were evaluated: magnetic stirring, set to medium rate, and vortex agitation. Moderate magnetic stirring was performed at position 2 on the stirrer’s 1–10 scale. As depicted in Figure 1g, agitation increased the absolute recoveries of the target compounds and was therefore selected as a better condition. For magnetic stirring, three elution times were investigated: 10, 15, and 20 min. According to the results, 15 min was selected as the optimal elution time for magnetic stirring, as there were no significant changes in recovery at 20 min. However, as the elution time was found to be relatively long, vortex agitation was investigated as a faster alternative, while also improving the operational practicality of the method. For vortex agitation, three elution times were evaluated: 1, 3, and 5 min. As shown in Figure 1g, 1 min of vortex agitation was enough to provide the highest recovery rates, while longer elution times resulted in analyte loss. Although magnetic stirring showed slightly higher absolute recoveries for most analytes, vortex agitation for 1 min was chosen as the optimal condition for the elution step, due to the significantly shorter elution time, introducing a greener and more practical approach.

The numerical data underlying Figure 1 are provided in the Supplementary Materials (Table S1). The final optimized FPSE protocol is described in Section 3.5.

### 2.2. Method Validation Results

The FPSE–HPLC–DAD method was validated in terms of linearity, sensitivity, selectivity, precision, and accuracy using spiked water samples, which were subjected to the optimized FPSE protocol, as described in Section 3.5.

**Figure 2:**
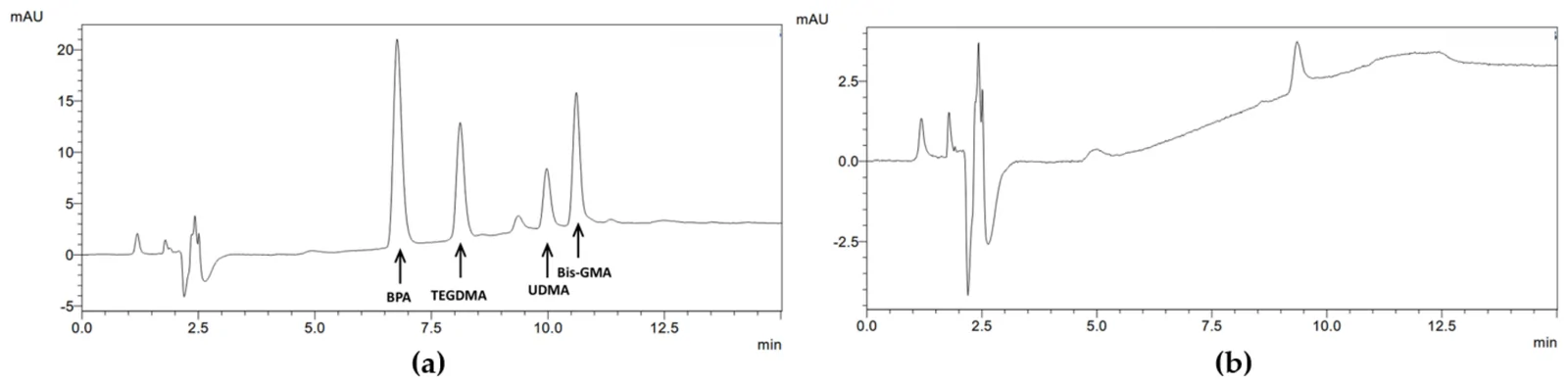
Chromatograms of (**a**) spiked 5 ng/μL water sample after the optimized FPSE procedure; and (**b**) blank water sample after the optimized FPSE procedure.

Linearity was evaluated covering the entire working range from 0.05 to 5 ng/μL for BPA, TEGDMA and Bis-GMA, and from 0.13 to 5 ng/μL for UDMA. The calibration curves showed good linearity for all target monomers, with coefficients of determination (R^2^) ranging from 0.9967 to 0.9995. The results from linear least squares regression analysis are presented in Table 2. The limits of detection (LODs) and quantification (LOQs) were 0.015 ng/μL and 0.05 ng/μL, respectively, for BPA, TEGDMA, and Bis-GMA, and 0.04 ng/μL and 0.13 ng/μL, respectively, for UDMA. The selectivity of the method was assessed by the analysis of blank water samples, which revealed absence of significant interferences. One peak between TEGDMA and UDMA at approximately 9.4 min appeared in every chromatogram but did not interfere with the separation and quantitation of the target monomers. Typical chromatograms of water spiked at 5 ng/μL and blank water samples, which underwent the FPSE sample preparation protocol, are presented in Figure 2. The method demonstrated satisfactory precision, with relative standard deviation (RSD) values lower than 7.7% for intra-day and lower than 9.5% for inter-day studies. Relative recovery values ranged from 93.9% to 100.8% (intra-day) and from 92.0% to 101.7% (inter-day) for all target analytes, indicating good accuracy. Method validation results for accuracy and precision are presented in Table 3.

### 2.3. Reusability

The reusability of the FPSE membrane is a significant advantage of the FPSE technique. It helps reduce the cost of the analysis, as the same membrane can be used for multiple samples, and enhances the environmental sustainability by reducing the need for new materials. The reusability of the sol–gel Si-PTHF membrane was evaluated by reusing the same FPSE membrane multiple times under the conditions of the optimized FPSE protocol. The results demonstrated that the membrane can be reused up to twelve times without a significant decrease in extraction efficiency (up to 10% of loss), as the recoveries of the target compounds remained high. Additionally, a carryover test was performed by immersing the FPSE membrane in ACN immediately after use, followed by analysis of the solution, in which no analyte peaks were detected.

### 2.4. Application to Real Sample

**Figure 3:**
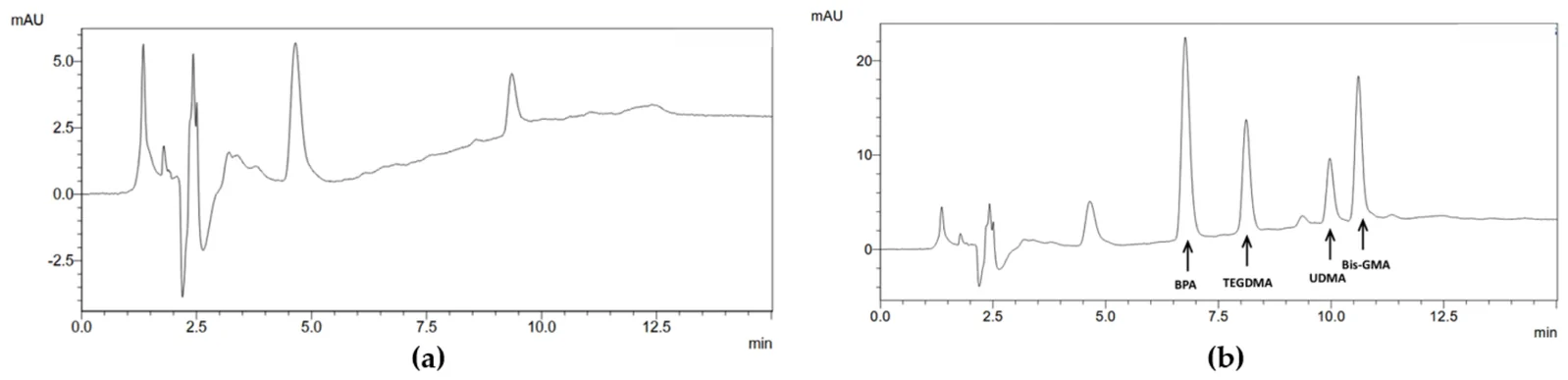
Chromatograms of the (**a**) dental wastewater sample and (**b**) spiked 5 ng/μL dental wastewater sample.

A real dental wastewater sample from the Dentistry Department of Aristotle University of Thessaloniki was analyzed using the aforementioned method. The sample was collected and stored in a glass container with a lid. Upon receipt, it was immediately centrifuged for 15 min to remove any suspended solid particles present. The supernatant then underwent sample preparation with the FPSE protocol and was analyzed by HPLC–DAD. In the chromatogram obtained (Figure 3a), no peaks were observed at the retention times of the four target compounds. Consequently, BPA, TEGDMA, UDMA, and Bis-GMA were not detected in the dental wastewater sample at concentrations above the method’s LODs.

Because the sample was centrifuged and filtered prior to FPSE analysis, the proposed method evaluates the filtered/dissolved fraction of dental wastewater rather than the total content of resin-derived compounds. Therefore, particle-bound analytes that may have been retained during pretreatment were not included in the analytical determination.

This result should not be interpreted as evidence that the targeted analytes are absent from dental wastewater. The wastewater sample that was analyzed was a pool of clinical wastewater samples collected from several drainage systems of dental units, and the actual concentration of the released compounds could be affected by the type and timing of the restorative procedures performed, by the amount of composite adjustment or removal, and by the degree of dilution of the wastewater. Furthermore, some of the resin-constituent compounds, especially those having a high molecular weight and hydrophobic properties, may be poorly soluble in the aqueous medium and be bound to the particles carrying the resin compounds. Previous studies have proven that the elution of monomers in aqueous media is compound dependent: the water solubility of TEGDMA is relatively good, but other larger and basic monomers demonstrate poor elution because of their size, chemical structure, and water insolubility. The release of resin-constituent compounds can also be time dependent, as certain compounds are released only in small amounts initially and increase with time [31]. As the sample was centrifuged and filtered prior to FPSE analysis, particle-bound analytes retained in the pretreatment procedure may have been lost. Thus, the actual example of the analysis of real wastewater shows the applicability of the proposed FPSE–HPLC–DAD method to analyze the dissolved fraction of dental wastewater, whereas the conclusions about the environmental presence of the compounds will need further sampling of wastewater obtained during different types of procedures conducted in different clinics and at various times, as well as future investigation of both dissolved and particulate fractions. For comparative evaluation, the corresponding chromatogram of the real sample is illustrated in Figure 3a, while the chromatogram of the spiked real sample at the concentration of 5 ng/μL is presented in Figure 3b.

Furthermore, to evaluate the accuracy and precision of the developed analytical method in real samples, spiked dental wastewater samples were prepared at the concentration levels of 0.5, 2, and 5 ng/μL. Samples were first centrifuged and then filtered using a 0.22 μm syringe filter to remove any remaining suspended particle. For each concentration level, three independent analyses were performed. According to the results presented in Table 4, relative standard deviation (RSD) values were lower than 7.6%, while relative recovery values ranged from 91.6% to 104.4%, confirming the suitability of the developed method for the analysis of real dental wastewater samples.

### 2.5. Evaluation of Method’s Green Character, Applicability, and Analytical Performance

A holistic approach to the evaluation of an analytical method is based on three distinct evaluation pillars, aimed at achieving sustainability, as described by the concept of white analytical chemistry (WAC) [32]. The first pillar addresses the greenness of the analytical method, the second focuses on its practicality, and the third concerns its analytical performance.

**Figure 4:**
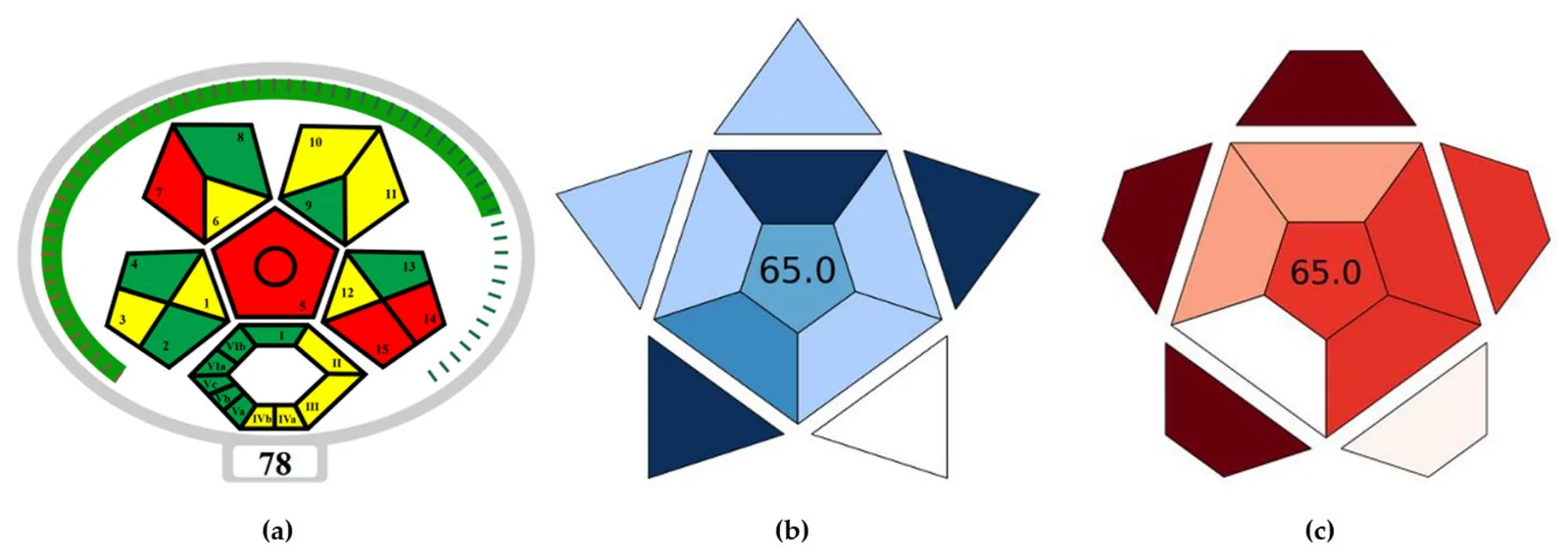
(**a**) ComplexMoGAPI, (**b**) BAGI, and (**c**) RAPI pictograms of the FPSE–HPLC–DAD method.

The greenness of an analytical method refers to the compliance with the principles of GAC and the assessment of the method’s environmental friendliness. To evaluate the green character of the method, the metric tool Complex Modified GAPI (ComplexMoGAPI) was used [33]. The FPSE–HPLC–DAD method received an overall score of 78, with the corresponding ComplexMoGAPI pictogram shown in Figure 4a, indicating a good level of greenness. The analytical method presents several advantages, such as low solvent consumption and simple sorbent preparation.

To evaluate the practicality and the applicability of the analytical method, the metric tool Blue Applicability Grade Index (BAGI) was used [34]. BAGI focuses on the practical aspects of the method, assessing parameters such as the number of analytes simultaneously determined, the required analytical instrumentation, and the automation degree. The BAGI pictogram for the FPSE–HPLC–DAD method is presented in Figure 4b, with the method’s score being 65, indicating good practicality and applicability in the analytical laboratory. The proposed method offers several important advantages, including the simultaneous determination of four compounds, the requirement of simple and commonly available instrumentation, the simultaneous analysis of multiple samples, the absence of a preconcentration step, and the small sample volume. However, its main disadvantage is the need for manual sample preparation and analysis.

In 2025, the Red Analytical Performance Index (RAPI) was introduced as a new tool for evaluating the validation criteria of an analytical method [35]. This metric tool allows easy evaluation and direct comparison of the analytical performance of multiple analytical methods. The RAPI score assigned to the FPSE–HPLC–DAD method is 65, indicating good analytical performance. The corresponding pictogram is shown in Figure 4c. The method’s repeatability and intermediate precision showed moderate performance, while reproducibility was not evaluated. The accuracy, recovery, and selectivity of the method were satisfactory, according to the tool’s criteria. Regarding the limit of detection, working range, and linearity, the method demonstrated maximum performance.

### 2.6. Comparison of the FPSE–HPLC–DAD Method with Other Reported Methods

To date, most studies have focused on assessing the release of BPA, TEGDMA, UDMA, and Bis-GMA from dental resin composites and evaluating their potential effects on patients, while there has been insufficient scientific research about the occurrence and the potential impact of these compounds on the environment. Due to the originality of the subject matter, only a limited number of previously reported studies on dental wastewater matrix were available for comparison. The available literature includes one study in which a protocol originally developed for human saliva matrix was applied to dental wastewater without validation [10], while another study introduced an analytical method, based on the SPE sample preparation technique, for the determination only of BPA [30] (Table 5).

The present study provides a validated, simple, and low-cost analytical method for the simultaneous determination of BPA, TEGDMA, UDMA, and Bis-GMA in dental wastewater. The method requires a small sample volume and uses the simple and widely available technique of HPLC–DAD, combined with a rapid FPSE sample preparation procedure, while providing satisfactory analytical performance. Moreover, there was no need for solvent evaporation and reconstitution.

## 3. Materials and Methods

### 3.1. Chemicals and Reagents

MeOH, ACN and H_2_O of HPLC grade were purchased from Chem-Lab (Zedelgem, Belgium). Sodium chloride (NaCl) was obtained from Sigma-Aldrich (St. Louis, MO, USA). Analytical-grade BPA, TEGDMA, UDMA, and Bis-GMA were supplied by Sigma–Aldrich-LLC (Taufkirchen, Germany). The five FPSE media evaluated in this study were fabricated using a sol–gel coating technology on hydrophilic cellulose fabric and were kindly provided by the Department of Chemistry and Biochemistry, Florida International University, Miami, FL, USA. Detailed preparation and characterization procedures are available in previous publications [29].

### 3.2. Preparation of Standards, Spiked, and Real Dental Wastewater Samples

Stock standard solutions (1000 ng/μL) of BPA, TEGDMA, UDMA, and Bis-GMA were prepared by dissolving the appropriate amount of each monomer in methanol. Working standard solutions were prepared by further dilution of each stock solution with methanol. All stock and working standard solutions remained stable for three months when stored at 4 °C. Spiked water samples were prepared by spiking HPLC-grade water with the appropriate volume of the standard solutions to obtain concentrations covering the entire linear range, from 0.05 to 5 ng/μL for BPA, TEGDMA and Bis-GMA, and from 0.13 to 5 ng/μL for UDMA. HPLC-grade water was used as a simplified blank matrix for the initial optimization and validation of the analytical procedure, because the target application involves an aqueous wastewater matrix and because a true blank dental wastewater sample free of the target analytes could not be reliably obtained. However, dental wastewater is a complex matrix that may contain saliva, suspended particulate matter, resin-derived particles, dental residues, and other organic or inorganic components. Therefore, water cannot fully reproduce the matrix effects of real dental wastewater. To further assess the applicability of the method to the target matrix, filtered dental wastewater samples were also spiked with standard solutions at final concentrations of 0.5, 2, and 5 ng/μL and analyzed under the optimized FPSE–HPLC–DAD conditions. Upon receipt from the School of Dentistry at Aristotle University of Thessaloniki, the dental wastewater sample was centrifuged for 15 min at 4000 rpm. Then, the supernatant was filtered through a 0.22 μm syringe filter to remove suspended solid particles that could clog the pores of the FPSE media and reduce extraction efficiency.

### 3.3. Collection of Dental Wastewater Sample

A pooled wastewater sample of dental origin was collected at the School of Dentistry, Aristotle University of Thessaloniki, Greece. The sample comprised wastewater collected after performing restorative dentistry treatment using resin-based composite materials. The sample was collected in a glass container with a lid to reduce the possibility of contaminating the sample with plastic components. Following collection of the sample, it was transported to the laboratory where it was analyzed right away. The sample was subjected to 15 min centrifugation at 4000 rpm and then the supernatant was filtrated through 0.22 μm syringe filter prior to FPSE. No chemical preservative was used before analysis.

### 3.4. Instrumentation and Chromatographic Conditions

An HPLC system consisting of an LC-20_AD_ pump, a DGU-20A online degassing unit, an SCL-10AL VP system controller, a CTO-6 A column oven and an SPD-M10A VP diode array detector (DAD) (Shimadzu, Kyoto, Japan) was employed for the chromatographic analysis. Sample injection was performed using a Rheodyne 7725i manual injector equipped with a 20 μL sample loop (Rheodyne, Rohnert Park, CA, USA). Chromatographic separation was achieved using a LiChrospher^®^ 100 Å RP-18e (5 µm, 250 × 4.0 mm) analytical column (Merck, Darmstadt, Germany), while data acquisition and instrument control were carried out using Shimadzu LC Solutions-LC software.

**Figure 5:**
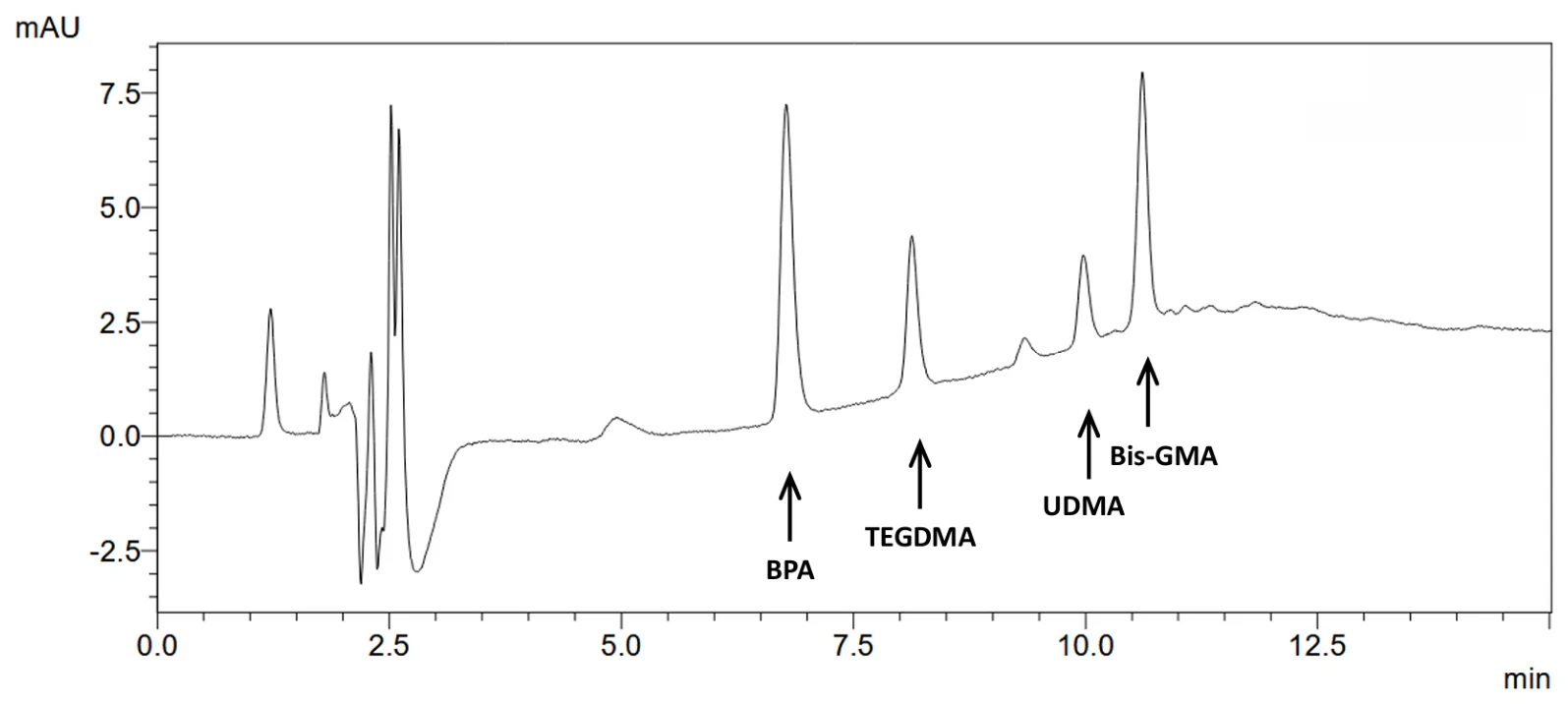
Chromatogram of the methanolic standard solution of the four compounds (1 ng/μL).

Various chromatographic conditions, including the mobile phase composition, elution mode, column temperature, and detection wavelength, were investigated by analyzing a standard mixture of the target analytes to determine the optimal HPLC conditions. A gradient elution using a mobile phase consisting of acetonitrile and water at a 1 mL/min flow rate was used for the separation of analytes. The gradient program started at 40% acetonitrile and increased to 80% (0–6 min), remained at 80% (6–12 min), returned to the initial composition of 40% acetonitrile (12–13 min), and was maintained until 15 min (13–15 min) for column re-equilibration. The column temperature was maintained at 40 °C. The detection, identification, and quantification of the target monomers were performed at a wavelength of 220 nm for all target analytes. Chromatographic analysis was completed within 15 min. The retention times of BPA, TEGDMA, UDMA, and Bis-GMA were 6.77, 8.12, 9.97, and 10.60 min, respectively, as shown in the chromatogram obtained from a 1 ng/μL methanolic working standard mixture, under optimal chromatographic conditions (Figure 5).

### 3.5. Fabric Phase Sorptive Extraction Protocol

**Figure 6:**
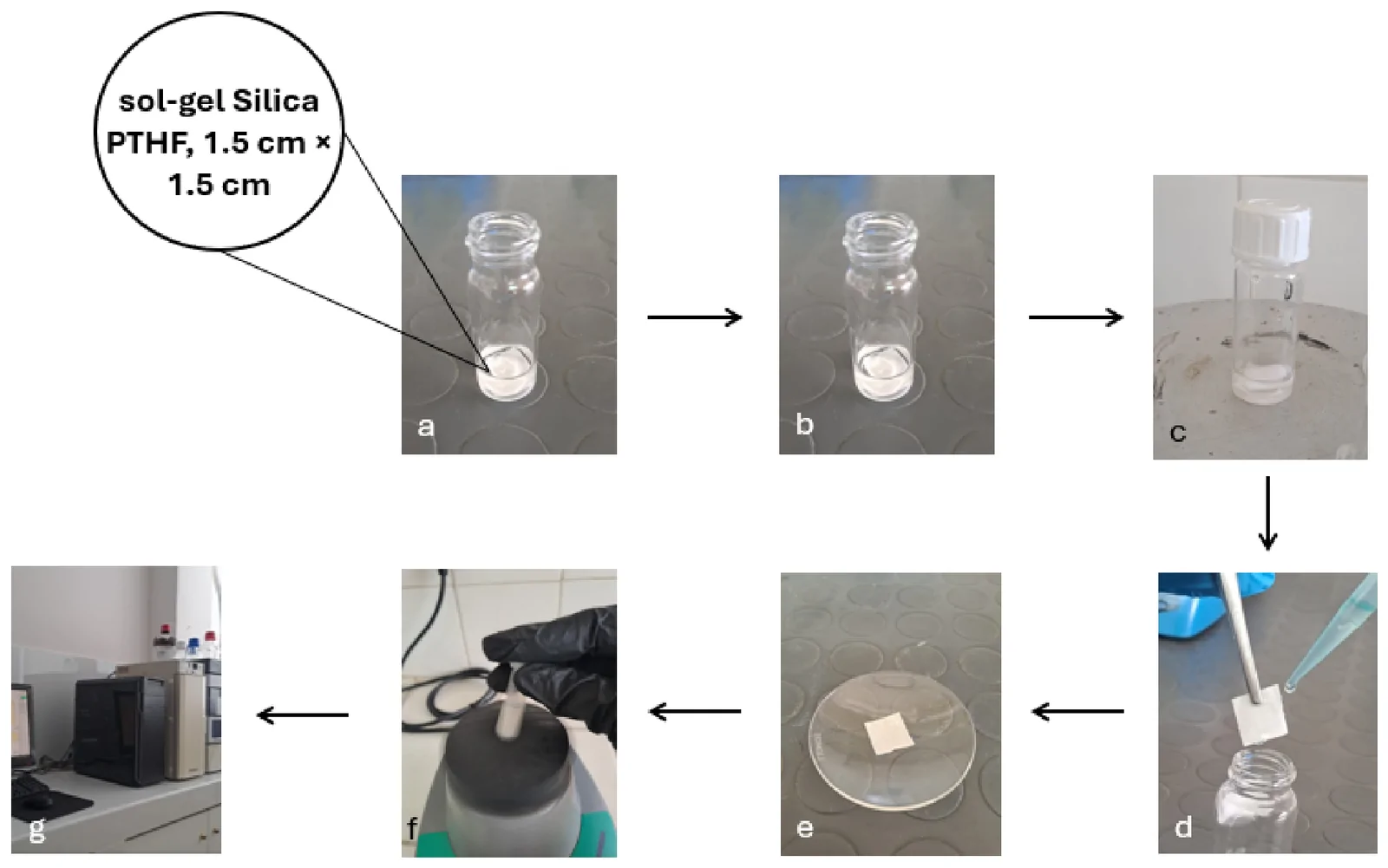
Schematic representation of the FPSE procedure: (**a**) Activation and cleaning step in 2 mL ACN:MeOH (5 min); (**b**) Washing step in 2 mL water (5 min); (**c**) Extraction step in 1 mL sample under magnetic stirring (20 min); (**d**) Rinsing of the FPSE membrane with water; (**e**) Drying of the FPSE membrane (3 min); (**f**) Elution step in 1 mL ACN under vortex (1 min); (**g**) Analysis by HPLC–DAD system.

The FPSE membrane utilized was sol–gel silica poly-tetrahydrofuran (sol–gel Si-PTHF) coated, with a square shape and a size of 2.25 cm^2^ (1.5 cm × 1.5 cm). The membrane was handled with tweezers to prevent any possible contamination. For the activation and cleaning step, the sol–gel Si-PTHF-coated membrane was immersed in 2 mL of acetonitrile–methanol (50:50, *v*/*v*) for 5 min. Subsequently, it was placed in 2 mL HPLC-grade water for 5 more minutes to remove the remaining organic solvents. For the extraction step, the cleaned FPSE membrane was transferred to a 5 mL screw-capped glass vial containing 1 mL of sample. A Teflon-coated magnetic stir bar was added to the vial, and the extraction was carried out in 20 min under moderate stirring. After extraction, the membrane was removed from the vial, rinsed with HPLC-grade water, and left to air dry for 3 min on a glass disk. For the elution step, the membrane was transferred to an Eppendorf tube containing 1 mL of acetonitrile as the elution solvent and was vortexed for just 1 min. The eluate was filtered through a syringe filter to remove any suspended particles, and was then injected directly into the HPLC–DAD system for analysis. The FPSE procedure is illustrated in Figure 6. After each extraction, the FPSE membrane was immersed in an acetonitrile–methanol (50:50 *v*/*v*) mixture to remove any residual compounds that may have remained on the sorbent material, left to air dry and stored in empty and clean vials until future use.

### 3.6. Optimization of FPSE Protocol

The FPSE protocol was optimized using the one-factor-at-a-time (OFAT) approach [36]. This approach was selected due to its simplicity and its ability to evaluate the effect of each experimental parameter. One experimental parameter varied at a time while all other parameters were kept constant. Once the optimal condition for each parameter was determined, it was maintained during the optimization of the next parameters.

Five different sorbent coatings on 100% cellulose fabric substrate were evaluated, namely, sol–gel silica-poly(tetrahydrofuran) (sol–gel Si-PTHF), sol–gel titania-poly(tetrahydrofuran) (sol–gel Ti-PTHF), sol–gel titania-poly(ethylene glycol)300 (sol–gel Ti-PEG300), sol–gel titania-carbowax 20M (sol–gel Ti-CW 20M), and sol–gel titania-poly(caprolactone)–polydimethylsiloxane–poly(caprolactone) (sol–gel Ti-PCAP-PDMS-PCAP). All critical parameters optimized included (a) membrane type, (b) membrane size (1–4 cm^2^), (c) extraction time (10–25 min), (d) stirring during extraction (without stirring, moderate stirring rate, and high stirring rate), (e) sample volume (1–2 mL), (f) ionic strength (0–10% NaCl), (g) elution solvent (MeOH, ACN, and MeOH: ACN (50:50, *v*/*v*)), (h) elution time (1–20 min), and (i) agitation during elution (without agitation, magnetic stirring, and vortex). The optimization was performed on water samples spiked with the target analytes at a concentration of 1 ng/μL, while all the optimization experiments were conducted in triplicate (*n* = 3). The % absolute recoveries of analytes were used as a selection criterion and were calculated as (peak area after extraction/peak area of the standard solution) × 100.

### 3.7. Method Validation

The FPSE–HPLC–DAD method was validated in terms of linearity, sensitivity, selectivity, accuracy, and precision. Linearity was evaluated by analyzing spiked water samples in triplicate (*n* = 3) within the working range from 0.05 to 5 ng/μL for BPA, TEGDMA and Bis-GMA, and from 0.13 to 5 ng/μL for UDMA. Calibration plots were obtained by plotting peak area versus analyte concentration. Linear least squares regression analysis was used to calculate slope, intercept and coefficient of determination (R^2^). The limits of detection (LODs) and quantification (LOQs) were estimated based on signal-to-noise (S/N) ratios of 3:1 and 10:1, respectively. The selectivity of the method for the target monomers was validated by the analysis of blank water samples. Accuracy and precision were studied by spiked water samples at three different concentrations (0.5, 2, and 5 ng/μL) in triplicate. Accuracy was evaluated as relative recovery (RR%), calculated as (measured concentration/spiked concentration) × 100. Precision was expressed as relative standard deviation (RSD%), calculated as (standard deviation/mean measured concentration) × 100. Intra-day repeatability was evaluated at the three concentration levels in triplicate (*n* = 3) within the same day, whereas inter-day precision was evaluated at the same concentration levels in triplicate over three subsequent days (*n* = 3 × 3). Moreover, membrane reusability was assessed by repeatedly using the same membrane in 12 extraction cycles. The membrane was considered reusable until the recovery fell below 90% of the initial value.

## 4. Conclusions

An environmentally friendly, practical and cost-effective analytical method was developed and validated for the simultaneous determination of BPA, TEGDMA, UDMA, and Bis-GMA, released from dental resin composites in dental wastewater, using an optimized FPSE protocol, followed by HPLC–DAD. This study aims to address the gap in the literature regarding the release of these monomers into the environment. To the best of the authors’ knowledge, this is the first validated analytical method for the simultaneous determination of these four target analytes in wastewater originating from dental units. Regarding the FPSE technique, significant parameters were optimized to increase extraction efficiency. The selected FPSE membrane was sol–gel Si-PTHF coated, with dimensions of 1.5 cm × 1.5 cm (2.25 cm^2^). Following the optimization, the method was validated in terms of linearity, sensitivity, selectivity, accuracy, and precision, showing satisfactory results. Moreover, the sol–gel Si-PTHF membrane was found to be reusable up to twelve times, contributing to the method’s sustainability and cost-effectiveness. The developed method was applied to the filtered/dissolved fraction of a real dental wastewater sample, demonstrating its applicability for the analysis of dissolved target compounds after sample pretreatment. Further studies using multiple wastewater samples and including both dissolved and particulate fractions are required before broader conclusions can be drawn regarding environmental monitoring.

## Figures and Tables

**Table 1:** Absolute recoveries (%) of FPSE membranes evaluated, including standard deviations (SDs).

	Absolute Recovery, AR% (n = 3)			
FPSE Membrane Type	BPA	TEGDMA	UDMA	Bis-GMA
Sol–gel Si-PTHF	61.2 ± 6.0	80.8 ± 3.9	73.1 ± 4.7	76.9 ± 12.0
Sol–gel Ti-PTHF	42.4 ± 2.8	57.0 ± 5.4	63.7 ± 6.0	73.3 ± 3.8
Sol–gel Ti-CW 20M	40.0 ± 0.5	31.6 ± 3.6	67.9 ± 3.3	73.8 ± 7.6
Sol–gel Ti-PCAP-PDMS-PCAP	-	-	-	-
Sol–gel Ti-PEG300	43.2 ± 1.0	47.2 ± 5.3	64.8 ± 6.5	70.4 ± 6.5

**Table 2:** Linearity data.

Analyte	Regression Equation	Linear Range (ng/μL)	R^2^	LOD (ng/μL)	LOQ (ng/μL)
BPA	y = 54013x − 99	0.05–5.0	0.9995	0.015	0.05
TEGDMA	y = 28341x + 30	0.05–5.0	0.9983	0.015	0.05
UDMA	y = 15804x − 1911	0.13–5.0	0.9967	0.04	0.13
Bis-GMA	y = 36842x − 646	0.05–5.0	0.9990	0.015	0.05

**Table 3:** Intra-day and inter-day accuracy and precision results.

Analyte	Added (ng μL^−1^)	Intra-Day (n = 3)			Inter-Day (n = 3 × 3)		
		Found ± SD (ng μL^−1^)	RSD%	RR%	Found ± SD (ng μL^−1^)	RSD%	RR%
BPA	0.5	0.50 ± 0.03	6.5	100.4	0.50 ± 0.02	4.0	100.2
	2	1.95 ± 0.07	3.4	97.6	1.92 ± 0.11	5.6	96.2
	5	4.95 ± 0.15	3.1	98.9	5.02 ± 0.17	3.5	100.4
TEGDMA	0.5	0.50 ± 0.00	0.8	99.7	0.49 ± 0.02	4.8	97.3
	2	1.90 ± 0.13	7.1	94.9	1.90 ± 0.13	6.6	94.8
	5	5.01 ± 0.14	2.8	100.2	5.06 ± 0.14	2.8	101.2
UDMA	0.5	0.50 ± 0.03	7.7	99.1	0.50 ± 0.03	7.1	99.3
	2	1.88 ± 0.08	4.3	93.9	1.84 ± 0.16	9.5	92.0
	5	5.04 ± 0.12	2.4	100.8	5.09 ± 0.10	2.1	101.7
Bis-GMA	0.5	0.48 ± 0.02	4.5	96.1	0.49 ± 0.01	3.1	97.5
	2	1.90 ± 0.14	7.3	95.1	1.90 ± 0.16	8.4	95.0
	5	5.01 ± 0.15	2.9	100.2	5.06 ± 0.13	2.7	101.2

**Table 4:** Accuracy and precision of the method in the spiked real sample (n = 3).

Analyte	Added (ng μL^−1^)	Found ± SD (ng μL^−1^)	RSD%	RR%
BPA	0.5	0.52 ± 0.02	3.3	104.4
	2	1.96 ± 0.04	2.3	97.8
	5	4.78 ± 0.05	1.1	95.6
TEGDMA	0.5	0.49 ± 0.04	7.6	98.0
	2	2.07 ± 0.04	1.8	103.3
	5	5.02 ± 0.12	2.3	100.3
UDMA	0.5	0.50 ± 0.00	1.3	100.7
	2	2.00 ± 0.09	4.5	100.1
	5	4.86 ± 0.10	2.0	97.1
Bis-GMA	0.5	0.50 ± 0.03	7.0	100.2
	2	1.95 ± 0.09	4.7	97.3
	5	4.58 ± 0.08	1.7	91.6

**Table 5:** Comparison of the proposed method with other reported methods on dental wastewater.

Analytes	Analytical Technique	Extraction Technique	Sample Volume	R (%)	RSD (%)	LOD (ng/μL)	LOQ (ng/μL)	Ref.
BPA	HPLC–FLD	SPE	5 mL	87.0–99.0%	-	0.001	0.005	[30]
BPA	HPLC–UV	FPSE	-	-	-	BPA: 0.0825	BPA: 0.25	[10]
TEGDMA						TEGDMA: 0.0825	TEGDMA: 0.25	
UDMA						UDMA: 0.165	UDMA: 0.5	
Bis-GMA						Bis-GMA: 0.165	Bis-GMA: 0.5	
BPA	HPLC–DAD	FPSE	1 mL	92.0–101.7%	<9.5%	BPA: 0.015	BPA: 0.05	**Current study**
TEGDMA						TEGDMA: 0.015	TEGDMA: 0.05	
UDMA						UDMA: 0.04	UDMA: 0.13	
Bis-GMA						Bis-GMA: 0.015	Bis-GMA: 0.05	

## Data Availability

The raw data supporting the conclusions of this article will be made available by the authors on request.

## Supplementary Materials

The following supporting information can be downloaded at: https://www.mdpi.com/article/10.3390/molecules31213873/s1, Table S1: Numerical data underlying Figure 1, expressed as absolute recoveries (%) for BPA, TEGDMA, UDMA, and Bis-GMA, including standard deviations (SD), for the optimization of (a) membrane size, (b) extraction time, (c) stirring during extraction, (d) sample volume, (e) ionic strength, (f) elution solvent, and (g) elution time and agitation.
