# Supplementary material for: Fabric Phase Sorptive Extraction Prior to HPLC–DAD Determination of Monomers Released from Dental Resin Composites in Dental Wastewater
Source: Molecules. 2026 Oct 27;31(21):3873. doi: 10.3390/molecules31213873 (PMC13765158; doi:10.3390/molecules31213873)
Supplement: Supplementary file 1 [file molecules-31-03873-s001.zip › molecules-4583059-supplementary.pdf]

Supplementary Materials

Fabric phase sorptive extraction prior to HPLC-DAD determination of monomers released from dental resin composites in dental wastewater

Ifigeneia Tragianni [1], Pavlos Voutsas [1], Natasa Kalogiouri [1], Abuzar Kabir [2],
Petros Mourouzis [3], Victoria Samanidou [1,*]

1 School of Chemistry, Laboratory of Analytical Chemistry, Aristotle University of Thessaloniki, Thessaloniki, Greece.
2 International Forensic Research Institute, Department of Chemistry and Biochemistry, Florida International University, Miami, Florida, USA.
3 School of Dental Tissues Pathology and Therapeutics, Division of Operative Dentistry, Faculty of Dentistry, Aristotle University of Thessaloniki, Thessaloniki, Greece.
* Correspondence: samanidu@chem.auth.gr

Table S1. Numerical data underlying Figure 1, expressed as absolute recoveries (%) for BPA, TEGDMA, UDMA, and Bis-GMA, including standard deviations (SD), for the optimization of: (a) membrane size, (b) extraction time, (c) stirring during extraction, (d) sample volume, (e) ionic strength, (f) elution solvent, and (g) elution time and agitation.

(a)

	**1 x 1 cm**	**1 x 1.5 cm**	**1.5 x 1.5 cm**	**2 x 2 cm**
**BPA**	67.2 ± 3.8	70.6 ± 4.1	82.4 ± 3.9	79.7 ± 4.1
**TEGDMA**	100.5 ± 6.3	87.8 ± 4.4	105.0 ± 5.0	92.0 ± 8.4
**UDMA**	89.2 ± 3.8	57.8 ± 6.6	81.9 ± 5.7	74.2 ± 5.3
**Bis-GMA**	74.5 ± 11.9	53.8 ± 3.2	85.3 ± 5.3	72.6 ± 5.5

(b)

	**10 min**	**15 min**	**20 min**	**25 min**
**BPA**	59.2 ± 4.3	60.8 ± 4.3	67.2 ± 3.8	67.2 ± 3.2
**TEGDMA**	80.4 ± 5.9	81.3 ± 6.7	100.5 ± 6.3	92.0 ± 7.3
**UDMA**	73.5 ± 9.9	75.6 ± 7.6	89.2 ± 3.8	86.8 ± 3.4
**Bis-GMA**	66.8 ± 5.6	67.3 ± 6.0	74.5 ± 11.9	61.9 ± 4.0

(c)

	**without stirring**	**moderate stirring rate**	**high stirring rate**
**BPA**	40.4 ± 4.1	84.1 ± 0.2	67.7 ± 13.7
**TEGDMA**	49.1 ± 0.6	90.3 ± 1.7	77.3 ± 14.7
**UDMA**	39.5 ± 0.1	79.9 ± 5.7	44.1 ± 11.5
**Bis-GMA**	42.5 ± 0.2	73.6 ± 5.4	44.4 ± 14.3

(d)

	**1 mL**	**2 mL**
**BPA**	82.4 ± 3.9	68.0 ± 4.9
**TEGDMA**	105.0 ± 5.0	75.9 ± 2.1
**UDMA**	81.9 ± 5.7	63.5 ± 2.2
**Bis-GMA**	85.3 ± 5.3	53.7 ± 9.2

(e)

	0% NaCl	5% NaCl	10% NaCl
**BPA**	82.4 ± 3.9	78.9 ± 5.9	82.0 ± 3.7
**TEGDMA**	105.0 ± 5.0	84.3 ± 6.3	84.6 ± 7.0
**UDMA**	81.9 ± 5.7	79.8 ± 8.8	77.1 ± 12.6
**Bis-GMA**	85.3 ± 5.3	57.6 ± 11.1	57.9 ± 16.0

(f)

	MeOH	MeOH:ACN (50:50 v/v)	ACN
**BPA**	68.7 ± 3.5	69.8 ± 7.3	67.2 ± 3.8
**TEGDMA**	88.5 ± 4.6	97.9 ± 12.4	100.5 ± 6.3
**UDMA**	72.1 ± 5.1	80.2 ± 12.5	89.2 ± 3.8
**Bis-GMA**	65.8 ± 6.7	66.9 ± 9.3	74.5 ± 11.9

(g)

	10 min / without stirring	10 min / stirring	15 min / stirring	20 min / stirring	1 min / vortex	3 min / vortex	5 min / vortex
**BPA**	61.2 ± 6.0	73.9 ± 6.1	75.3 ± 4.5	77.9 ± 11.7	72.8 ± 5.8	68.7 ± 3.5	68.5 ± 2.3
**TEGDMA**	80.8 ± 3.9	88.0 ± 5.8	96.4 ± 4.8	96.5 ± 12.3	91.3 ± 11.3	88.5 ± 4.6	87.1 ± 6.0
**UDMA**	73.1 ± 4.7	76.3 ± 6.8	78.8 ± 1.0	80.3 ± 10.7	82.9 ± 12.3	72.1 ± 5.1	76.7 ± 8.1
**Bis-GMA**	76.9 ± 12.0	74.2 ± 7.9	71.1 ± 3.7	74.3 ± 10.2	65.8 ± 2.8	65.8 ± 6.7	70.6 ± 5.6
